# Association of specific chromosome alterations with tumour phenotype in posterior uveal melanoma

**DOI:** 10.1054/bjoc.1999.0923

**Published:** 2000-01-18

**Authors:** K Sisley, M A Parsons, J Garnham, A M Potter, D Curtis, R C Rees, I G Rennie

**Affiliations:** Institute for Cancer Studies, University of Sheffield Medical School, Sheffield, UK; Department of Ophthalmology and Orthoptics, Sheffield University, Hallamshire Hospital, Sheffield, UK; Langhill, Centre for Human Genetics, Sheffield, UK; Department of Life Sciences, Clifton Campus, Nottingham Trent University, Nottingham, UK

**Keywords:** cytogenetics, translocation, uveal melanoma, phenotype

## Abstract

Posterior uveal melanomas have recurrent alterations of chromosomes 1, 3, 6 and 8. In particular, changes of chromosomes 3 and 8 occur in association, appear to characterize those tumours with a ciliary body component, and have been shown to be of prognostic significance. The relevance of other chromosome alterations is less certain. We have performed cytogenetic analysis on 42 previously untreated primary posterior uveal melanomas. Of interest was the observation that as tumour size increased the involvement of specific chromosome changes, and the amount of chromosome abnormalities likewise increased. Loss, or partial deletions, of the short arm of chromosome 1 were found to associate with larger ciliary body melanomas; typically, loss of the short arm resulted from unbalanced translocations, the partners of which varied. Trisomy of chromosome 21 occurred more often in ciliary body melanomas, whilst rearrangements of chromosomes 6 and 11 were primarily related to choroidal melanomas. Our results imply that alterations of chromosome 1 are important in the progression of some uveal melanomas, and that other chromosome abnormalities, besides those of chromosomes 3 and 8, are associated with ocular tumours of particular locations. © 2000 Cancer Research Campaign


*British Journal of Cancer* (2000) **82**(2), 330–338
© 2000 Cancer Research Campaign
Article no. bjoc.1999.0923

### Association of specific chromosome alterations with tumour phenotype in posterior uveal melanoma

**K Sisley[1,2], MA Parsons[2], J Garnham[3], AM Potter[3], D Curtis[3], RC Rees[4] and IG Rennie[2]**

[1]Institute for Cancer Studies, University of Sheffield Medical School, Sheffield, UK; [2]Department of Ophthalmology and Orthoptics, Sheffield University, Hallamshire Hospital, Sheffield, UK; [3]Langhill, Centre for Human Genetics, Sheffield, UK; [4]Department of Life Sciences, Clifton Campus, Nottingham Trent University, Nottingham, UK

**Summary** Posterior uveal melanomas have recurrent alterations of chromosomes 1, 3, 6 and 8. In particular, changes of chromosomes 3 and 8 occur in association, appear to characterize those tumours with a ciliary body component, and have been shown to be of prognostic significance. The relevance of other chromosome alterations is less certain. We have performed cytogenetic analysis on 42 previously untreated primary posterior uveal melanomas. Of interest was the observation that as tumour size increased the involvement of specific chromosome changes, and the amount of chromosome abnormalities likewise increased. Loss, or partial deletions, of the short arm of chromosome 1 were found to associate with larger ciliary body melanomas; typically, loss of the short arm resulted from unbalanced translocations, the partners of which varied. Trisomy of chromosome 21 occurred more often in ciliary body melanomas, whilst rearrangements of chromosomes 6 and 11 were primarily related to choroidal melanomas. Our results imply that alterations of chromosome 1 are important in the progression of some uveal melanomas, and that other chromosome abnormalities, besides those of chromosomes 3 and 8, are associated with ocular tumours of particular locations. © 2000 Cancer Research Campaign

**Keywords:** cytogenetics; translocation; uveal melanoma; phenotype

The eye is the commonest site for non-cutaneous melanomas, accounting for approximately 80% of such lesions (Scotto et al, 1976). Ocular melanomas principally arise from the uveal tract, which comprises the iris, ciliary body and the choroid, the highest incidences are for the posterior (ciliary body and choroid) melanomas, with an annual frequency, in America, of 5–7 cases per million population (Egan et al, 1988; Rennie, 1991). A genetic predisposition has been suggested to be amongst the predisposing risk factors for this disease, with at least 40 families showing increased susceptibility (Canning and Hungerford, 1988; Singh et al, 1996), and recent evidence has implied that a relationship may exist with breast cancer (Wooster et al, 1995).

The cytogenetic alterations of the majority of solid malignancies are poorly understood, with solid tumours comprising approximately 27% of all reported cytogenetic analyses and, of these, less than 1% relate to melanomas (Mitelman, 1994). Posterior uveal melanomas are relatively atypical amongst adult solid tumours, because they are highly amenable to chromosome analysis and often possess only minimal cytogenetic alterations (Prescher et al, 1990, 1995; Sisley et al, 1990, 1992; Horsman and White, 1993; Wiltshire et al, 1993; Singh et al, 1994). This propensity has permitted the identification of consistent anomalies of chromosomes 3, 6 and 8; in particular, loss of chromosome 3 is associated with alterations of chromosome 8, principally in the form of an isochromosome 8q, and such abnormalities appear to be correlated with those tumours with a ciliary body component (Sisley et al, 1990, 1992; Prescher et al, 1992, 1996; Dahlenfors et al, 1993; Horsman and White, 1993; Singh et al, 1994). Ciliary body melanomas are known to have an adverse prognosis (McLean et al, 1977), and the close association of chromosomes 3 and 8 with this phenotype has led to the suggestion, and confirmation, that the presence of such chromosome alterations identify uveal melanoma patients with significantly reduced survival (Prescher et al, 1996; Sisley et al, 1997). Although uveal melanomas do not usually demonstrate high levels of chromosome aberrations, alterations of other chromosomes, such as those of chromosomes 1, 11, 21 and the Y chromosome, also occur with a reasonable consistency (Prescher et al, 1990, 1995; Sisley et al, 1990, 1992; Horsman and White, 1993; Gordon et al, 1994; Singh et al, 1994; Speicher et al, 1994); but it is unclear whether these changes associate with specific stages of tumour development, or with tumour sub-groups. In this report, we examined the specific involvement of individual chromosomes in a series of 42 primary uveal melanomas. Patients were referred with previously untreated uveal melanomas, during an 8-year period, to a national centre based at the department of Ophthalmology and Orthoptics, Sheffield University. Cytogenetic analysis of some patients had been reported formerly (Sisley et al, 1990, 1992; Tappin et al, 1996), and the significance of chromosomes 3 and 8 changes already documented (Sisley et al, 1997).

### MATERIALS AND METHODS

### Clinical details

Informed patient consent and ethical approval were obtained prior to commencing the study. A series of 42 formally untreated patients with posterior uveal melanomas (ciliary body and

*Received 3 September 1998*
*Accepted 30 March 1999*
*Correspondence to:* K Sisley, Institute for Cancer Studies, Sheffield University Medical School, Beech Hill Road, Sheffield, South Yorkshire, S10 2RX, UK

**Table 1** Clinicopathological details of the patients

| Case no | Sex | Tumour location and cell type | Diameter and volume in (mm) |
|---|---|---|---|
| Som 10 | Male | Choroid (C), Spindle cell (S) | 12.45/929.53 |
| Som 11 | Female | Cilary Body (CB), Mixed cell (M) | 11.1/1352.75 |
| Som 14 | Male | C, M | 15.25/769.37 |
| Som 17 | Male | C, M | 13.5/1025.83 |
| Som 22 | Male | Cilary body/choroid (CB/C), M | 12.7/1419.15 |
| Som 27 | Male | C, Epithelioid (E) | 11.8/769.64 |
| Som 30 | Male | CB/C | – |
| Som 35 | Male | CB, E | 12/398.13 |
| Som 37 | Female | CB/C, M | – |
| Som 40 | Female | C, M | 11/590.97 |
| Som 42 | Male | C, M | 15.1/2043.15 |
| Som 44 | Female | CB/C, M | 15.3/1518.99 |
| Som 47 | Male | C, M | 13.25/466 30 |
| Som 52 | Female | CB/C, M | 17.45/1664.67 |
| Som 53 | Male | CB/C, M | 15.6/2897.72 |
| Som 57 | Female | CB/C, M | 14.7/2226.14 |
| Som 59 | Male | C, M | 17.1/2282.73 |
| Som 60 | Male | C, M | 9.55/825.28 |
| Som 61 | Male | C, M | 13.45/962.84 |
| Som 62 | Male | CB/C, E | 18.05/2168.57 |
| Som 63 | Male | C, S | 16.3/723.1198 |
| Som 95 | Male | CB/C, M | 21.75/3939.70 |
| Som 96 | Female | CB, M | 16.8/3292.50 |
| Som 98 | Male | C, S | 12.05/1349.02 |
| Som 99 | Female | CB, S | 12/387.81 |
| Som 100 | Female | CB, E | 13.5/1172.09 |
| Som 112 | Male | CB/C, S | 19/2605.95 |
| Som 113 | Male | CB/C, M | 19.65/2918.02 |
| Som 115 | Male | CB, E | 10.8/500.22 |
| Som 118 | Male | C, S | 16.4/1477.20 |
| Som 119 | Male | C, M | 24/4041.91 |
| Som 120 | Male | CB, M | 6.3/392.77 |
| Som 122 | Male | CB, M | 12.65/1492.11 |
| Som 124 | Male | CB/C, S | 22.2/5241.26 |
| Som 125 | Female | C, M | 19.1/2239.61 |
| Som 126 | Male | CB/C, M | 16.75/2937.64 |
| Som 128 | Female | C, S | 15.45/2478.55 |
| Som 134 | Male | C, S | 10.3/756.65 |
| Som 135 | Male | CB, M | 19.4/1657.42 |
| Som 136 | Male | C, S | 11.3/530.60 |
| Som 137 | Female | CB/M | 16.2/1580.36 |
| Som 140 | Female | CB/S | 13.6/1836.91 |

choroid) were examined during the period 1987–1995. The majority of patients were treated by enucleation (removal of the eye), although six patients underwent local resection of the primary tumour. The cohort consisted of 29 males and 13 females, ranging in age from 43 to 90 years (median 66 years). Estimation of tumour size was conducted preoperatively by B scan ultrasonography (Cooper Vision), and the longest tumour diameter in contact with the sclera was determined (Ltd). All melanomas were categorized histopathologically according to the AFIP system of classification for uveal melanoma (Spencer, 1986). The clinicopathological details of the previously unreported patients are presented in Table 1.

### Cytogenetic analysis

Uveal melanoma biopsies were processed immediately upon resection as previously reported (Sisley et al, 1990, 1997). Tumour cultures were continually assessed, and when sufficient growth had been procured, cells were harvested and chromosome preparations made and banded (Sisley et al, 1990), with abnormalities recorded in accordance with international guidelines (ISCN, 1991).

### Statistical analysis

Statistical association was assessed by constructing 2×2 contingency tables of the variables. Statistical significance was evaluated using Pearson $\chi^2$ and Fishers' Exact test with a *P*-value of $< 0.05$ taken to indicate significance.

### RESULTS

Cytogenetic analysis and the clinical and histopathological details of 18 patients has been reported previously. Cases Som 10, 11, 17, 22, 30 and 35 were presented in Sisley et al (1990), cases Som 36, 37, 47, 52, 53, 57, 59, 60, 61, 62 and 63, were detailed in Sisley et al (1992) and cases Som 95 and 140 reported in Tappin et al (1996). The significance of chromosome 3 and 8 abnormalities in the series of 42 patients has been considered separately (Sisley et al, 1997). Cytogenetic analysis from the uveal melanomas is detailed in Table 2 and the clinicopathological details are presented in Table 1.

The results of cytogenetic analysis for all 42 cases was examined. The incidence for involvement of individual chromosomes in numerical and structural changes was ascertained as a percentage of tumours possessing such abnormalities (results not shown). In a given tumour, if aneuploidy of both homologues was present, or if both experienced structural rearrangements, the occurrence was only recorded once. From the study the commonest chromosome abnormalities were confirmed to be loss of chromosome 3 (21 tumours, 50% of melanomas) and the Y chromosome (16 tumours, 38% of melanomas), and structural alterations of chromosomes 1, 6 and 8 (13, 16 and 21 tumours representing 30%, 38% and 50% of melanomas respectively). The distribution of breakpoints for structural alterations in the series was determined (Figure 1), and although most chromosomes experienced some rearrangement, the occurrence of breakpoints was not random. Chromosome 8 was the most heavily implicated in structural rearrangements, with breakpoints concentrated at the centromeric region, whilst both arms of chromosome 6 were equally represented. Breakpoints on chromosome 1 were predominantly at the centromeric region, and, in common with other breakpoints on chromosome 1p, produced an effectual deletion of the short arm, often as a result of unbalanced translocations (Figure 2). Also of interest was the apparent hotspot at chromosome 11q23 (Figure 1).

To associate chromosome abnormalities with phenotypic features, the incidence of numerical and structural alterations was determined in relation to tumour location, size and cell type (Figures 3 and 4). The significance of the specific changes of chromosomes 3 (loss) and 8 has been previously recounted (Sisley et al, 1997), and will not be considered in this report. Structural alterations of chromosome 6 (p and q) were found in both ciliary body (8/24 tumours) and choroid melanomas (8/18 tumours) ($P = 0.46$). Trisomy of chromosome 21 occurred more frequently in ciliary body melanomas (6/24) although this did not achieve statistical significance ($P = 0.23$). Similarly, chromosome 1 p deletions were found predominantly in ciliary body melanomas (11/24); this was statistically significant ($P = 0.016$). Abnormalities of chromosome

© 2000 Cancer Research Campaign

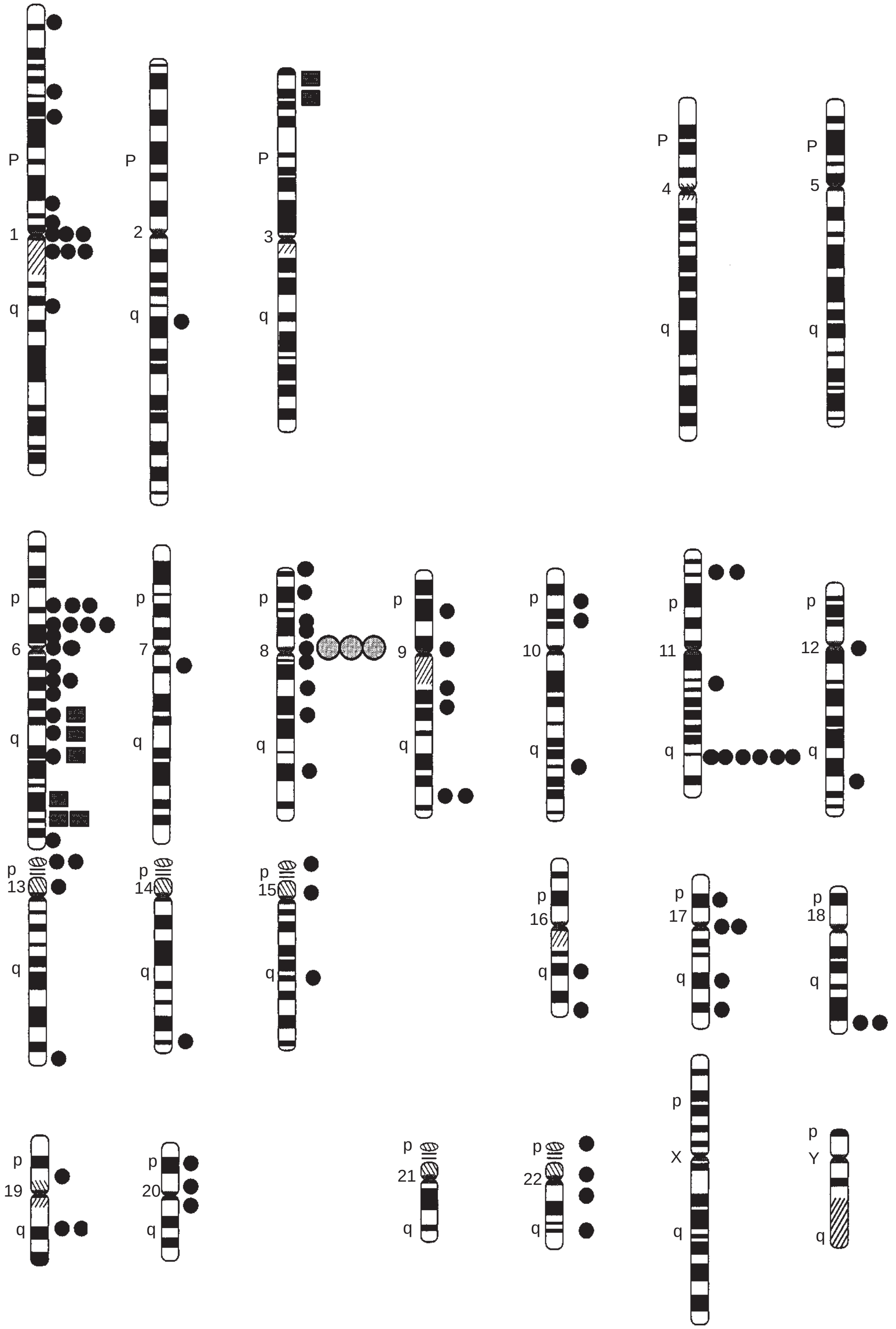

**Figure 1** Idiogram showing distribution of breakpoints for structural alterations in the series. ● denotes a tumour with a breakpoint in this region, ● denotes five tumours with breakpoints ■ denotes a tumour with a possible breakpoint in this region.

11 occurred more frequently in choroidal tumours, again this failed to achieve statistical significance ($P = 0.10$).

From comparison of the cytogenetic changes with increasing tumour size, it was observed that an increase in tumour size, whether in the diameter, thickness or volume, was associated with an increase in the number of chromosomes partaking in numerical changes (Figure 4A). Tumours greater than 15 mm in diameter possessed approximately three times the numbers of total numerical abnormalities compared with those of less than 15 mm diameter, although a corresponding increase for structural alterations was not observed (Figure 4B). When individual homologues were considered, abnormalities of chromosomes 1, 3, 6, 8,

© 2000 Cancer Research Campaign

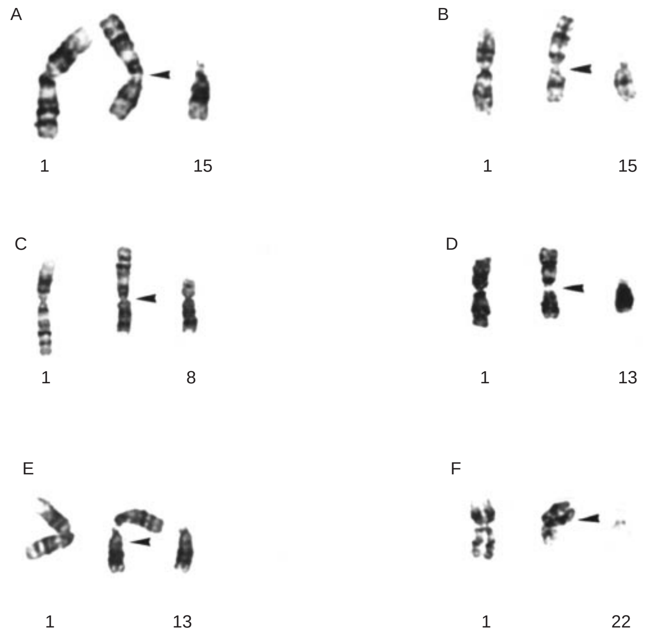

**Figure 2** Unbalanced translocations affecting chromosome 1, producing effectual deletions of the short arm. Abnormal chromosomes are indicated by arrowheads. (**A**) Case Som 11, der(15)t(1;15)(q11;p13), first reported in Sisley et al (1990); (**B**) Case Som 112, der(15)t(1;15)(q11;p13); (**C**) case Som 113, der(1)t(1;8)(q10;q10); (**D**) case Som 115, der(13)t(1;13)(q11;p13); (**E**) case Som 120, der(13)t(1;13)(q11;p13); (**F**) case Som 137, der(22)t(1;22)(q11;p11)

9, 10, 17 and Y were found to be more common in the larger tumours (Figure 4), although involvement was not significant for any chromosome ($P$ = 0.31, $P$ = 0.18, $P$ = 0.50, $P$ = 0.37, $P$ = 0.053, $P$ = 0.07, $P$ = 0.15 and $P$ = 0.51 respectively). No specific trends for chromosome abnormalities could be related to cell type (data not shown).

### DISCUSSION

The association of chromosomes 3, 6 and 8 changes with posterior uveal melanoma has been clearly documented (Prescher et al, 1990, 1995; Sisley et al, 1990, 1992; Dahlenfors et al, 1993; Horsman and White, 1993; Wiltshire et al, 1993; Singh et al, 1994). Equally well recognized is the relationship between chromosome 6 and cutaneous melanoma (Ray et al, 1996), and the observation that similar alterations of chromosome 6 occur in uveal melanoma (Prescher et al, 1990, 1995; Sisley et al, 1990; Horsman and White, 1993; Wiltshire et al, 1993; Singh et al, 1994). It is unclear as to which chromosome changes may be considered to be related to a general pathology of cell type, such as neural crest derivation, or even melanoma associated changes, and what changes, if any, depict differences in those melanomas arising in sites other than the skin. Indeed recent evidence suggests that amongst uveal melanomas (ciliary body, choroid and iris) those melanomas arising in the iris have cytogenetic abnormalities different to those of the posterior uvea (White et al, 1995; Sisley et al, 1998), and as iris melanomas are by comparison relatively benign (Sunba et al, 1980), it is possible that these differences may account for variations in aggressive behaviour.

It is of interest that as the tumour enlarges there is a demonstrable increase in the numerical changes, whilst structural alterations show no such pattern. Greater aneuploidy may either be representative of specific essential changes associated with tumour progression, or, with the exception of chromosome 3 loss, indicate unrelated ‘background noise’ illustrative of the presumably advanced nature of these tumours (Johansson et al, 1996). Cytogenetic abnormalities can be segregated into balanced and unbalanced aberrations, according to their ultimate effect on the genetic constitution (Johansson et al, 1996). Under this model balanced structural abnormalities, such as translocations or inversions, result in gene rearrangements and can be considered as primary, whereas numerical changes, trisomies and monosomies, produce gross genetic imbalances and would be considered secondary (Johansson et al, 1996). In uveal melanoma most alterations yield unbalanced rearrangements and would therefore fall into the category of secondary, possibly unrelated changes (Johansson et al, 1996). It is also important to consider the potential implications of the mechanics behind these abnormalities. Numerical changes may simply arise as the by-product of poorly regulated cell division, comparable to the increased genetic instability associated with *p53* or *BUB1* mutant cells (Agapova et al, 1996; Cahill et al, 1998), and therefore occur more frequently in the larger uveal melanomas as a result of other, perhaps undetected, genetic abnormalities. Structural alterations require DNA breakage and reunion, and in uveal melanoma they occur without particular regard to increasing tumour size, with the possible exceptions of rearrangements of chromosomes 9 and 10 which are found in cutaneous and some uveal melanomas (Magauran et al, 1994; Healy et al, 1995; Ohta et al, 1996). It is possible that structural abnormalities could conceivably represent a more specific mechanism than for most numerical changes; regardless of whether they transpire to be primary or secondary aberrations.

In uveal melanoma certain alterations, particularly those of chromosomes 3 and 8, are known to be more common in tumours arising from select locations (Sisley et al, 1990, 1992; Prescher et al, 1992, 1996; Dahlenfors et al, 1993; Horsman and White, 1993; Singh et al, 1994), and the results of this study, in combination with previous cytogenetic analysis, would suggest that this classification can be extended to include other alterations. In ciliary body melanomas, or those of a mixed location, structural abnormalities of chromosomes 1 and 8, and aneuploidy of chromosomes 3 and 21, are more frequently observed (Sisley et al, 1990, 1992; Dahlenfors et al, 1993; Horsman and White, 1993; Wiltshire et al, 1993; Singh et al, 1994; Prescher et al, 1995). In choroid melanomas, or again those of a mixed location, rearrangements of chromosomes 6 and 11 are more likely (Sisley et al, 1990, 1992; Dahlenfors et al, 1993; Horsman and White, 1993; Singh et al, 1994; Prescher et al, 1995). An interesting paradox can be seen when observing mixed ciliary body/choroid melanomas, as they appear to combine chromosome aberrations from both localities. In these mixed melanomas abnormalities of chromosomes 1, 3, 6, 8, 11 and 21 are all found, although at the other extremes, changes of chromosomes 1, 3, 8 and 21 are infrequent amongst those melanomas entirely derived from the choroid, whilst equally rare are rearrangements of chromosomes 6 and 11 in purely ciliary body-derived melanomas. It is unclear why these mixed melanomas possess abnormalities of both locations, but it is possible that environmental factors may play a role. This association with tumour location may explain why chromosome alterations are predictive of survival. Ciliary body melanomas have a worse prognosis (McLean et al, 1977), and recent evidence

*© 2000 Cancer Research Campaign* 

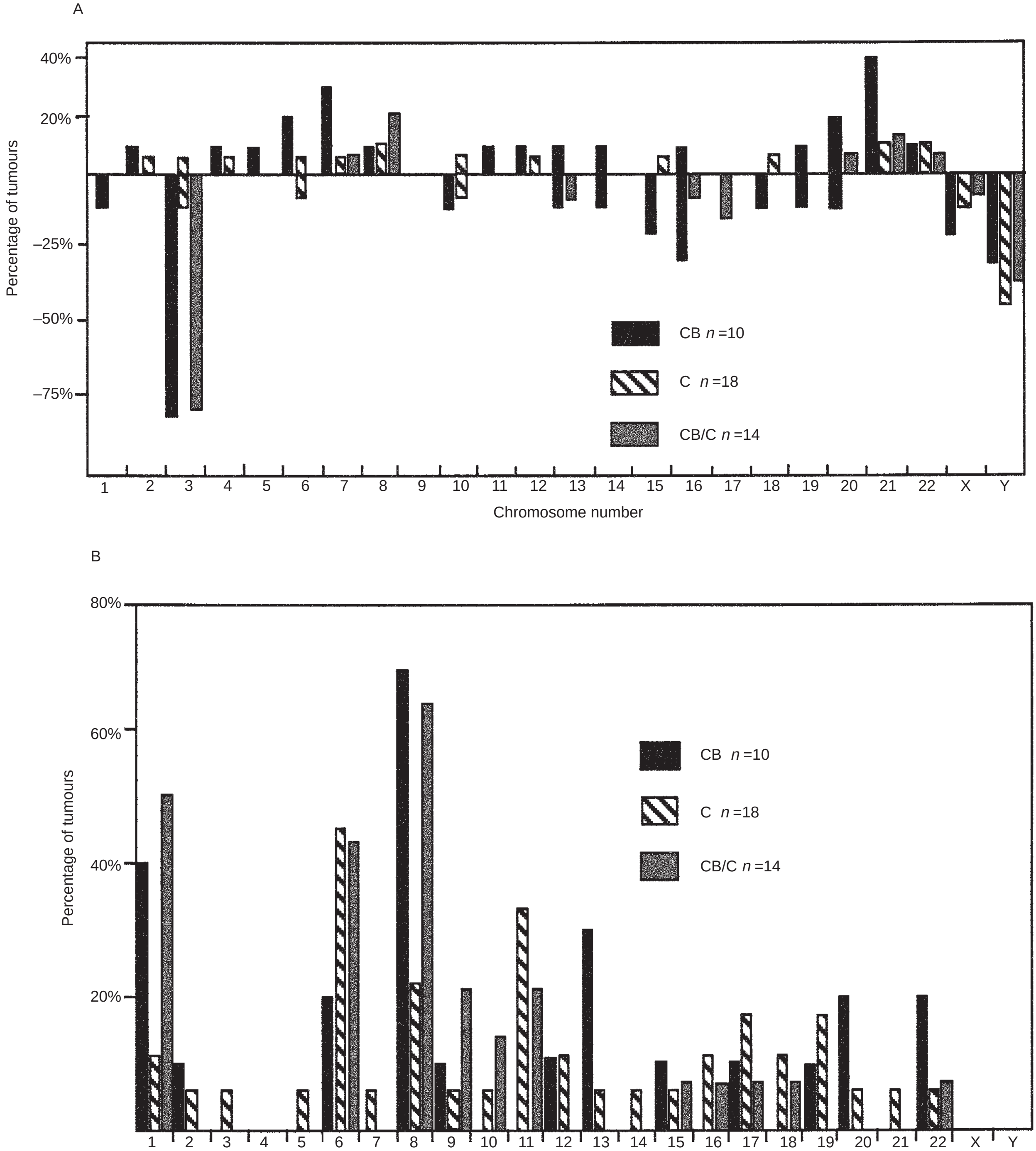

**Figure 3** The percentage of tumours with numerical (**A**) and structural (**B**) alterations of individual chromosomes related to tumour location. C = choroid melanomas, CB = ciliary body melanomas, CB/C = mixed site melanomas. (**A**) The loss of a chromosome is shown below the line, and the gain of a chromosome above suggests that abnormalities of chromosomes 3 and 8 identify a poor outcome, whilst those of chromosome 6, more common in choroid melanomas, are a good prognostic indicator (Prescher et al, 1996; Sisley et al, 1997; White et al, 1998). At least in terms of their cytogenetic alterations it would appear that these two melanomas have different genetic backgrounds, leading to speculation that in some way the locality itself dictates the genetic changes required for the tumour to progress, a suggestion perhaps compounded by the observation that the relatively benign iris melanomas have different changes (White et al, 1995; Sisley et al, 1998).

Through comparison of tumour enlargement with chromosome changes, it is apparent that certain aberrations become more frequent as the tumour progresses, including those of chromosomes 1, 3, 6, 8, 11 and Y, already identified as non-randomly involved. In addition trisomy of chromosome 21 and structural

© 2000 Cancer Research Campaign

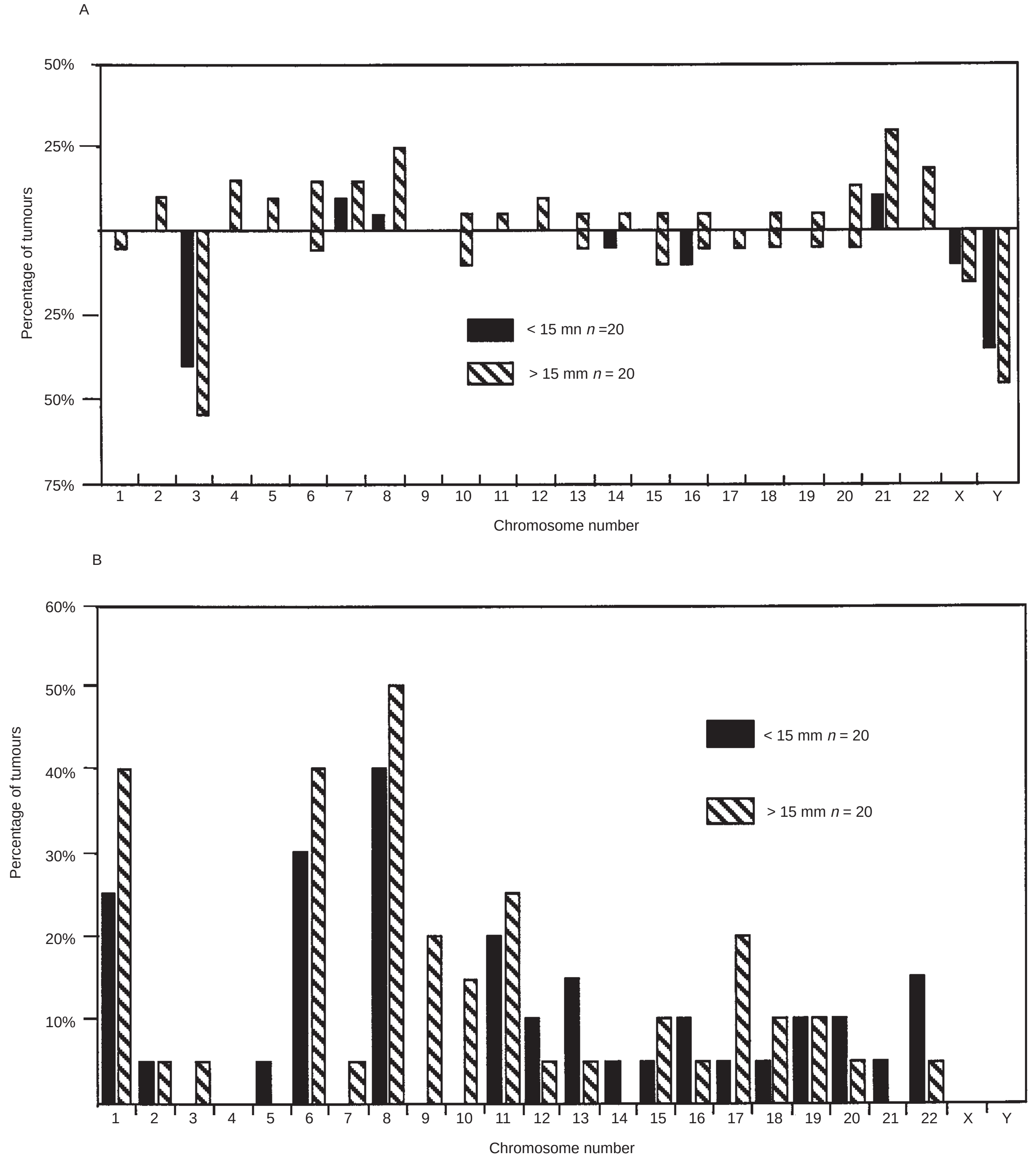

**Figure 4** The involvement of chromosomes in relation to increasing tumour size. (**A**) The percentage of tumours with numerical changes in tumours of above (> 15 mm) and below (< 15 mm) 15 mm diameter; (**B**) the percentage of tumours with structural alterations when compared to tumour diameter alterations of chromosomes 9, 10 and 17 also occur (Figure 4). Deletions of chromosomes 9, 10 and 17 are found in cutaneous melanomas (Healy et al, 1995), and uveal melanomas have also been shown to have loss of regions on chromosome 9 (Magauran et al, 1994; Ohta et al, 1996). As changes of chromosomes 1, 3, 8 and 21 are more common to melanomas with a ciliary body component (Figure 3), and because of their progressive acquisition, the sequence of cytogenetic alterations for ciliary body melanomas could be proposed as, monosomy chromosome 3 (Prescher et al, 1994), followed by additional 8q (often as an isochromosome), which is subject to amplification (Sisley et al, 1997), possibly accompanied at some later point by deletions of chromosome 1p and/or trisomy 21. In choroid melanomas the situation is less apparent, although the common involvement of chromosomes 6 and 11 alterations would suggest they may play a role. Since chromosome 6 rearrangements were detected in more

*© 2000 Cancer Research Campaign* 

**Table 2** Cytogenetic analysis of the tumours from patients with primary posterior uveal melanoma

| Case no | Karyotype |
|---|---|
| Som 10 | 7 Cells 46, XY |
| | 4 Cells 46,XY,der(11)t(6;11)(p21;p15) |
| Som 11 | 6 Cells 44,XX,–1,–3,+i(8)(q10),–14,der(15)t(1;15)(q11;p13) |
| | 4 Cells 44,XX,–1,–3,+i(8)(q10),–14,der(15)t(1;15)(q11;p13),del(20),(q11) |
| | 2 Cells 45,XX,–1,–3,+i(8)(q10),–14,der(15)t(1;15)(q11;p13), del(20)(q11),+mar |
| Som 14 | 4 Cells 46,X,–Y,der(15)t(6;15)(p12;q22),+mar,inc |
| Som 17 | 11 Cells 46,XY |
| Som 22 | 8 Cells 46,X,–Y,del(1)(p12p32),+7,add(22)(p13),add(22)(p13) |
| | 5 Cells 46,X,–Y,del(1)(p12p32),add(6)(q22),+7,add(22)(P13),add(22)(P13) |
| Som 27 | 4 Cells 47,XX,–3,+add(8)(p11),del(11)(q23),+mar,inc |
| Som 30 | 8 Cells 44,XY,–3,–6,i(8)(q10),+i(8)(q10),der(9)t(6;9)(p12;q21),–17 |
| | 5 Cells 43,XY,–3,–6,i(8)(q10),+i(8)(q10),der(9)t(6;9)(p12;q21),–16,–17 |
| Som 35 | 4 Cells 46,X,–Y,–3,i(6)(p10),+7,i(8)(q10),+i(8)(q10),–16,+21 |
| | 3 Cells 47,X,–Y,–3,i(6)(p10),+7,i(8)(q10),+i(8)(q10),+21,del(22)(q11) |
| | 5 Cells 46,X,–Y,–3,i(6p),+7,i(8)(q10),der(8;12)(q10;q10),+21 |
| Som 37 | 14 Cells 46,XX, |
| | 2 Cells 46,XX,–3,i(6)(p10),i(8)(q10),+i(8)(q10) |
| Som 40 | 4 Cells 47,X,–X,add(8)p12),+2mar,inc |
| Som 42 | 5 Cells 46,XY,del(6)(q14q15),add(17)(p12q24),+mar,inc |
| Som 44 | 3 Cells 45,XX,–3,del(6)(q?24q?25),+i(8)(q10),–17,inc |
| Som 47 | 27 Cells 46,XY,der(6)t(6;6)(q27;p12),del(11)(q23),add(12)(q24),add(14)(q32), add(18)(q23),add(19)(q13),add(22))(q13) |
| Som 52 | 11 Cells 46,XX,t(1;10)(p13;q24),–3,i(8)(q10),+i(8)(q10),del(11)(q23) |
| | 9 Cells 46,XX,t(1;10)(p13;q24),–3,+i(8)(q10),del(11)(q23) |
| | 6 Cells 45,XX,t(1;10)(p13;q24),–3,+i(8)(q10),del(11)(q23) |
| Som 53 | 17 Cells 44,XY,–3,add(6)(q13–14),–9,der(17)t(9;17)(q10;q10) |
| | 5 Cells 43,XY,–3,add(6)(q13–14),–9,–13,der(17)t(9;17)(q10;q10) |
| | 4 Cells 44,XY,–3,add(6)(q13–14),–9,–13,der(17)t(9;17)(q10;q10)+mar |
| Som 57 | 14 Cells 46,XX |
| | 3 Cells 45,XX,–3,i(8)(q10) |
| Som 59 | 10 Cells 49,XY,+8,der(13)t(6;13)(p12;q34),+der(13)t(6;13)(p12;q34),+21 |
| | 8 Cells 45,X,–Y |
| | 4 Cells 46,XY,der(13)t(6;13)(p12;q34) |
| | 3 Cells 46,XY |
| | 2 Cells 47,X,–Y,+6,+22 |
| Som 60 | 13 Cells 46,XY |
| | 6 Cells 45,X,–Y |
| Som 61 | 9 Cells 46,XY |
| Som 62 | 10 Cells 45,X,–Y,–3,+8 |
| | 4 Cells 45,X,–Y,–3,+8,del(11)(q13q13) |
| | 4 Cells 45,X,–Y,–3,+i(8)(q10),del(11)(q13q13) |
| Som 63 | 14 Cells 46,XY |
| | 5 Cells 46,XY,del(6)(q21q21) |
| | 4 Cells 46,XY,del(6)(q21q21),del(7)(q11q11) |
| | 3 Cells 47,XY,+5,del(6)(q21q21),del(7)(q11q11) |
| | 3 Cells 45,X,–Y |
| | 2 Cells 48,XY,+4,+5,del(6)(q21q21),del(7)(q11q11) |
| | 2 Cells 46,XY,–6,del(6)(q21q21),+10 |
| Som 95 | 6 Cells 44,X,–Y,der(1)t(1;8)(q10;q10),–3 |
| Som 96 | 6 Cells 46,XX |
| | 3 cells 72<3n>,XXX,–1,+2,–3,+6,+7,+8,+11,+12,–15,+16,–18,–19,+20 |
| Som 98 | 5 cells 45,X,–Y,del(11)(q23),inc |
| Som 99 | 2 cells 46,XX |
| Som 100 | 2 cells 46,XX |
| Som 112 | 7 cells 46,X,–Y–1,–3,+i(8)(q10),der(15)t(1;15)(q11;p13),+20,+21 |
| | 4 cells 47,X,–Y,i(1)(q10),–3,+8,+21,+22 |
| | 3 cells 48,X,–Y,–3,+8,+20,+21,+mar |
| | 3 cells 45,X,–Y,–1,–3,+i(8)(q10),der(15)t(1;15)(q11;p13),+21 |
| | 2 cells 48,X,–Y,–3,+i(8)(q10),der(15)t(1;15)(q11;p13),+20,+21,+mar |
| Som 113 | 10 cells 44,X,–Y,der(1)t(1;8)(q10;q10),–3 |
| | 2 cells 45,X,–Y,der(1)t(1;8)(q10;q10),–3,+21 |
| | 3 cells 44,X,–Y,der(1)t(1;8)(q10;q10),–3,del(11)(q?) |
| Som 115 | 5 cells 46,XY,–1,–3,del(6)(q?16q?22),+i(8)(q10),+i(8)(q10), der(13)t(1;13)(q11;p13),add(20)(p12) |
| Som 118 | 30 cells 46–54,X,–Y,+2[2],der(3)t(1;3)(q23;p24–25)[7],+4[4],–6[30],+7[3], +8[3],add(9)(q34)[11],add(9)(p12)[5],–10[10],add(10)(p12)[12], del(11)(q23)[3],+12[3],add(12)(p?)[2],+15[4],add(16)(q24)[8], +18[17],del(18)(p?)[5],add(19)(q13)[30],+21[3], +22[3], +1–5mar[cp30] |

*© 2000 Cancer Research Campaign*

**Table 2** Continued

| Case no | Karyotype |
|---|---|
| Som 119 | 5 cells 45,X,–Y |
| Som 120 | 3 cells 44,X,–Y,–1,–3,+i(8)(q10),der(13)t(1;13)(q11;p13)<br>4 cells 46,X,–Y,–1,–3,+i(8)(q10)×3,der(13)t(1;13)(q11;p13)<br>3 cells 94<4n>,XX,–Y,–Y,–1,–1,–3,–3,+i(8)(q10)×6,der(13)t(1;13)(q11;p13)×2, +21,+21 |
| Som 122 | 16 cells 44–45,X,–Y,[16],–3[16],i(8)(q10)[2],i(8)(q10),+i(8)(q10)[3],i(8)(q10), +i(8)(q10)×2[5],+i(8)(q10)×2[3],+i(8)(q10)×3[3],–13[6], add(13)(p11)[7],–16[12][cp16] |
| Som 124 | 10 cells 46,XY,del(1)(p?),add(6)(q21–25),add(9)(q34), der(10)(t(8;10)(q11;p13),der(18)t(6;18)(p21;q23) |
| Som 125 | 6 cells 48,X,–X,del(1)(p36),+del(1)(p36),–3,i(8)(q10),+i(8)(q10)×2, +der(8)t(8;8)(p23;q23)<br>4 cells 48,X,–X,del(1)(p36),+del(1)(p36),–3,i(8)(q10),+i(8)(q10)×2, +der(8)t(8;8)(p23;q23),i(17)(q10) |
| Som 126 | 10 cells 46,XY |
| Som 128 | 20 cells 46,XX,der(6)t(6;8)(q16;q13),der(11)t(6;11)(p21;q23), add(11)(p15),der(20)t(6;20)(p11;p11) |
| Som 134 | 8 cells 45,X,–Y<br>2 cells 46,XY |
| Som 135 | 7 Cells 73–75<3n>,X,–X[7],–X[7],del(2)(q?)[3],–3[7],+4[7],+5[7],+6[2],+7[4], i(8)(q10)[7],+i(8)(q10)[7],+13[2],+14[2],–15[2],–16[5], +19[2],–20[3],+20[2],+21[5],+22[4][cp7] |
| Som 136 | 9 Cells 46,XY<br>5 Cells 46,XY,del(2)(q22),add(5)(p?),del(6)(q?),add(16)(q?),add(17)(q?), add(19)(p?)<br>2 Cells 46,XY,del(2)(q22),add(5)(p?),del(6)(q?),add(16)(q?),add(17)(q?), add(19)(p?),del(21)(q?) |
| Som 137 | 2 Cells 44,X,-X,–1,–3,i(8)(q10),der(8)t(8;8)(p22;q21),del17(q21q22), der(19)t(9;19)(q13;p13),der(22)t(1;22)(q11;p11),+1mar<br>11 Cells 45,X,-X,–1,–3,i(8)(q10),der(8)t(8;8)(p22;q21),del17(q21q22), der(19)t(9;19)(q13;p13),+21,der(22)t(1;22)(q11;p11),+1mar<br>2 Cells 44,X,-X,–1–3,i(8)(q10),der(8)t(8;8)(p22;q21),–10,del17(q21q22), der(19)t(9;19)(q13;p13),+21,der(22)t(1;22)(q11;p11),+1mar |
| Som 140 | 4 Cells 44–46,X,-X[14],add(1)(p31)[5],–3[14],+8[14],add(16)(q21)[7], +mar1[5],+mar2[4][cp14] |

melanomas, it may be reasonable to expect that such changes occur prior to those of chromosome 11, of which breakpoints at, or around, 11q23 appear to be particularly implicated.

In uveal melanoma there is a remarkable consistency for reported structural aberrations (Prescher et al, 1990, 1995; Sisley et al, 1990, 1992; Dahlenfors et al, 1993; Horsman and White, 1993; Wiltshire et al, 1993; Singh et al, 1994), which in some instances includes both the selection of translocation partners and the location of breakpoints (Figures 1 and 2). In this study the frequent structural rearrangements of chromosome 1 are particularly interesting in this respect. These exchanges appear to be non-random yielding consistent genetic imbalances (deletions of 1p), perhaps important to the development, in particular the progression, of mainly ciliary body melanomas. Similar changes are also a common event in cutaneous melanomas, possibly associated with tumour progression, and several genes have been implicated (Dracopoli et al, 1989). In posterior uveal melanoma there appears to be a surprising affinity for certain translocation partners of chromosome 1 (Figure 2) particularly amongst the D Group, with reported cases detailing repeated involvement of chromosomes 8, 13, and 15 in comparable translocations with the same, or similar, breakpoints implicated (Prescher et al, 1990; Sisley et al, 1990, 1992; Horsman and White, 1993; Tappin et al, 1996). This reproducibility suggests an underlying mechanism in which regions containing highly repetitive DNA sequences are strategically implicated. An analogous situation is perhaps found in breast cancer, where a reproducible translocation affecting chromosomes 1 and 16 has been observed (Kokalj-Vokac et al, 1993). The breakpoints were found to span the heterochromatic regions, with the significant event considered to be the ensuing imbalance (Kokalj-Vokac et al, 1993). In posterior uveal melanoma in addition to the net imbalance, the preferential affiliation for repetitive sequences, without a prerequisite for pairing with the same homologue, may suggest other motives, perhaps related to deficiencies in DNA repair (Surralles et al, 1997). Whether this selection is essential or coincidental awaits disclosure.

Cytogenetic analysis of posterior uveal melanoma appears to provide valuable insights into the necessary genetic alterations related to its development and progression. This study would suggest that in addition to changes of chromosome 3 and 8, alterations of chromosomes 1 and 21 correlate with ciliary body melanomas, whereas in choroid melanomas abnormalities of chromosomes 6 and 11 are more frequent. It is also of interest that structural rearrangements of chromosome 1 appear to associate with tumour progression and in particular the consistency of breakpoints involved in these alterations may help to identify the underlying genes responsible for such behaviour.

*© 2000 Cancer Research Campaign*

### ACKNOWLEDGEMENTS

This work has been supported by grants from the Yorkshire Cancer Research Campaign (grant number S 253), the Medical Research Council (grant number G9106078CA), and the Macmillan Fund. We would like to thank Janet White, Robin Farr and Rhona Jacques for their assistance in the preparation of this work.

### REFERENCES

Agapova LS, Ilyinskaya GV, Turovets NA, Ivanov AV, Chumakov PM and Kopnin BP (1996) Chromosome changes caused by alterations of p53 expression. *Mutagenesis* **354**: 129–138

Cahill DP, Lengauer C, Yu J, Riggins GJ, Willson JKV, Markowitz SD, Kinzler KW and Vogelstein B (1998) Mutations of mitotic checkpoint genes in human cancers. *Nature* **392**: 300–303

Canning CR and Hungerford J (1988) Familial uveal melanoma. *Br J Ophthalmol* **72**: 241–243

Dahlenfors R, Tornqvist G, Wettrell K and Mark J (1993) Cytogenetical observations in nine ocular malignant melanomas. *Anticancer Res* **13**: 1415–1420

Dracopoli NC, Harnett P, Bale SJ, Stanger BZ, Tucker MA, Houseman DE and Kefford RF (1989) Loss of alleles from the distal short arm of chromosome 1 occurs late in melanoma tumour progression. *Proc Natl Acad Sci USA* **86**: 4614–4618

Egan KM, Seddon JM, Glynn RJ, Gragoudas ES and Albert DM (1988) Epidemiologic aspects of uveal melanoma. *Surv Ophthalmol* **32**: 239–257

Gordon KB, Thompson CT, Char DH, O'Brien JM, Kroll S, Ghazvini S and Gray JW (1994) Comparative genomic hybridization in the detection of DNA copy number abnormalities in uveal melanoma. *Cancer Res* **54**: 4764–4768

Healy E, Rehman I, Angus B and Rees JL (1995) Loss of heterozygosity in sporadic primary cutaneous melanoma. *Genes Chromosom Cancer* **12**: 152–156

Horsman DE and White VA (1993) Cytogenetic analysis of uveal melanoma. Consistent occurrence of monosomy 3 and trisomy 8q. *Cancer* **71**: 811–819

ISCN (International Standing Committee on Human Cytogenetic Nomenclature) (1991) *Guidelines for cancer cytogenetics, supplement to an international system for human cytogenetic nomenclature*, F Mitelman, (ed). S Karger: Basel

Johansson B, Mertens F and Mitelman F (1996) Primary vs. secondary neoplasia associated chromosomal abnormalities – balanced rearrangements vs. genomic imbalances? *Genes Chromosomes Cancer* **16**: 155–163

Kokalj-Vokac N, Alemeida A, Gerbault-Seureau M, Malfoy B and Dutrillaux B (1993) Two color FISH characterization of i(1q) and der(1;16) in human breast cancer cells. *Genes Chromosomes Cancer* **7**: 8–14

McLean MIW, Foster WD and Zimmerman LE (1977) Prognostic factors in small malignant melanomas of choroid and ciliary body. *Arch Ophthalmol* **95**: 48–58

Magauran RG, Gray B and Small KW (1994) Chromosome 9 abnormality in choroidal melanoma. *Am J Ophthalmol* **117**: 109–111

Mitelman F (1994) Database of cancer cytogenetics: an overview. In: *Catalog of Chromosome Aberrations in Cancer*, Mitelman F (ed), pp. ix–xviii. Wiley Liss: New York

Ohta M, Berd D, Shimizu M, Nagai H, Cotticelli MG, Mastrangelo M, Shields JA, Shields CL, Croce CM and Huebner K (1996) Deletion mapping of chromosome region 9p21–p22 surrounding the CDKN2 locus in melanoma. *Int J Cancer* **65**: 762–767

Prescher G, Bornfeld N and Becher R (1990) Non-random chromosomal abnormalities in primary uveal melanoma. *J Natl Cancer Inst* **82**: 1765–1769

Prescher G, Bornfeld N, Horsthemke B and Becher R (1992) Chromosomal aberrations defining uveal melanoma of poor prognosis. *Lancet* **339**: 691–692

Prescher G, Bornfeld N and Becher R (1994). Two subclones in a case of uveal melanoma. Relevance of monosomy 3 and multiplication of chromosome 8q. *Cancer Genet Cytogenet* **77**: 144–2146

Prescher G, Bornfeld N, Friedrichs W, Seeber S and Becher R (1995) Cytogenetics of twelve cases of uveal melanoma and patterns of nonrandom anomalies and isochromosome formation. *Cancer Genet Cytogenet* **80**: 40–46

Prescher G, Bornfeld N, Hirche H, Horsthemke B, Jockel KH and Becher R (1996) Prognostic implications of monosomy 3 in uveal melanoma. *Lancet* **347**: 1222–1225

Ray ME, Su YA, Meltzer PS and Trent JM (1996) Isolation and characterization of genes associated with chromosome 6 mediated tumor suppression in human malignant melanoma. *Oncogene* **12**: 2527–2533

Rennie IG (1991) Diagnosis and treatment of ocular melanomas. *Br J Hosp Med* **46**: 144–156

Scotto J, Fraumeni JF and Lee JAH (1976) Melanomas of the eye and other non-cutaneous sites: epidemiologic aspects. *J Natl Cancer Inst* **56**: 489–491

Singh AD, Boghosian-Sell L, Kishore KW, Wary KK, Shields CL, De Potter P, Donoso LA, Shields JA and Cannizzaro LA (1994) Cytogenetic findings in primary uveal melanoma. *Cancer Genet Cytogenet* **72**: 109–115

Singh AD, Shields CL, De Potter P, Shields JA, Trock B, Cater J and Pastore D (1996) Familial uveal melanoma clinical observations of 56 patients. *Arch Ophthalmol* **114**: 392–399

Sisley K, Rennie IG, Cottam DW, Potter AM, Potter CW and Rees RC (1990) Cytogenetic findings in six posterior uveal melanomas: involvement of chromosomes 3, 6 and 8. *Genes Chromosomes Cancer* **2**: 205–209

Sisley K, Cottam DW, Rennie IG, Parsons MA, Potter AM, Potter CW and Rees RC (1992) Non-random abnormalities of chromosomes 3, 6, and 8 associated with posterior uveal melanoma. *Genes Chromosomes Cancer* **5**: 197–200

Sisley K, Rennie IG, Parsons MA, Jacques R, Hammond DW, Bell SM, Potter AM and Rees RC (1997) Abnormalities of chromosomes 3 and 8 in posterior uveal melanoma correlate with prognosis. *Genes Chromosomes Cancer* **19**: 22–28

Sisley K, Brand C, Parsons MA, Maltby E, Rees RC and Rennie IG (1998) Cytogenetics of iris melanomas: disparity with other uveal melanomas. *Cancer Genet Cytogenet* **101**: 128–133

Speicher MR, Prescher G, du Manoir S, Jauch A, Horsthemke B, Bornfeld N, Becher R and Cremer T (1994) Chromosomal gains and losses in uveal melanoma detected by comparative genomic hybridization. *Cancer Res* **54**: 3817–3823

Spencer (1986) *Ophthalmic Pathology. An Atlas and Textbook Vol. 3* WB Saunders: Philadelphia

Sunba MSN, Rahi AHS and Morgan G (1980) Tumours of the anterior uvea I. Metastasizing malignant melanoma of the iris. *Arch Ophthalmol* **98**: 82–85

Surralles J, Darroudi F and Natarajan T (1997) Low level of DNA repair in human chromosome 1 heterochromatin. *Genes Chromosomes Cancer* **20**: 173–184

Tappin MJ, Parsons MA, Sisley K, Rees RC and Rennie IG (1996) Two cases of double melanoma of the uvea. *Eye* **10**: 600–602

White VA, Horsman DE and Rootman J (1995) Cytogenetic characterization of an iris melanoma. *Cancer Genet Cytogenet* **82**: 85–87

White VA, Chambers JD, Courtright PD, Chang WY and Horsman DE (1998) Correlation of cytogenetic abnormalities with the outcome of patients with uveal melanoma. *Cancer* **83**: 354–359

Wiltshire RN, Elner VM, Dennis T, Vine AK and Trent JM (1993) Cytogenetic analysis of posterior uveal melanoma. *Cancer Genet Cytogenet* **66**: 47–53

Wooster R, Bignell G, Lancaster J, Swift S and Seal S (1995) Identification of the breast cancer susceptibility gene BRCA 2. *Nature* **378**: 789–792

 *© 2000 Cancer Research Campaign*
